# Mitophagy and Mitochondrial Dysfunction in Cancer

**DOI:** 10.1146/annurev-cancerbio-030419-033405

**Published:** 2019-11-06

**Authors:** Kay F. Macleod

**Affiliations:** The Ben May Department for Cancer Research, University of Chicago, Chicago, Illinois 60637, USA

**Keywords:** mitophagy, Parkin, PINK1, BNIP3, NIX, mitochondrial depolarization, mitochondrial fission, DRP1, electron transport chain, reactive oxygen species, ferroptosis, DNA damage, NAD^+^, MiDAS, inflammasome, cGAS-STING

## Abstract

The process of mitophagy, in which mitochondria are selectively turned over at the autophagolysosome, plays a central role in both eliminating dysfunctional mitochondria and reducing mitochondrial mass as an adaptive response to key physiological stresses, such as hypoxia, nutrient deprivation, and DNA damage. Defects in mitophagy have been linked to altered mitochondrial metabolism, production of excess reactive oxygen species and ferroptosis, heightened inflammasome activation, altered cell fate decisions, and senescence, among other cellular consequences. Consequently, functional mitophagy contributes to proper tissue differentiation and repair and metabolic homeostasis, limiting inflammatory responses and modulating tumor progression and metastasis. This review examines the major pathways that control mitophagy, including PINK1-dependent mitophagy and BNIP3/NIX-dependent mitophagy. It also discusses the cellular signaling mechanisms used to sense mitochondrial dysfunction to activate mitophagy and how defective mitophagy results in deregulated tumor cell growth and cancer.

## MAINTAINING THE INTEGRITY OF DYNAMIC MITOCHONDRIA AS METABOLIC SIGNALING HUBS

Mitochondria, known as the powerhouses of the cell, are double-membraned organelles that evolved from endosymbiotic Alphaproteobacteria with their own genome and capacity to generate ATP through respiration; oxidize fatty acids; provide numerous metabolic intermediates essential for nucleotide, amino acid, and lipid biosynthesis; synthesize iron-sulphur complexes; and sequester calcium and other ions (Pagliarini & Rutter 2013). Mitochondrial integrity prevents programmed cell death (Gao et al. 2019, Vyas et al. 2016), while mitochondrial production of ROS (reactive oxygen species), acetyl CoA (coenzyme A), and other signaling intermediates regulates gene expression and cell fate determination (Matilainen et al. 2017, Sena & Chandel 2012).

Cellular mechanisms have evolved to maintain mitochondrial integrity and to preserve the strategic role of mitochondria in cell growth (D’Amico et al. 2017). Such mechanisms include the ability to undergo fission and fusion to modulate the efficiency of mitochondrial metabolite transport and to partition damaged mitochondrial DNA (mtDNA) (Mishra & Chan 2014). Mitochondria also dynamically control cristae structures, densities of electron chain supercomplexes, and rates of uncoupling to burn or conserve energy as needed (Liesa & Shirihai 2013). The mitochondrial unfolded protein response (UPR^mt^) is activated in response to amino acid deprivation and other stresses that cause an imbalance in the production of nuclear-encoded versus mitochondrial-encoded mitochondrial proteins that would otherwise disrupt mitochondrial function (D’Amico et al. 2017). Critically, cells regulate overall mitochondrial mass as a means to adapt to stress by modulating rates of mitochondrial biogenesis (Ploumi et al. 2017) and inducing mitochondrial turnover via mitophagy, which is the focus of this review.

Mitochondrial function is subverted in a multitude of ways during tumorigenesis, including through altered signaling to promote mitochondrial biogenesis (Vyas et al. 2016), induction of DRP1 (dynamin-related protein 1)-dependent fission (Kashatus et al. 2015, Serasinghe et al. 2015), acquired resistance to apoptosis (Vyas et al. 2016), metabolic reprogramming to benefit cell growth (Kimmelman & White 2017, Zong et al. 2016), selection for specific mtDNA mutations (Zong et al. 2016), and elevated mitochondrial ROS production (Sena & Chandel 2012), among other effects as reviewed extensively elsewhere (Vyas et al. 2016, Zong et al. 2016). The role of mitophagy in cancer has been examined as a consequence of inhibiting general autophagy in tumor models, where accumulation of defective mitochondria with altered metabolic and signaling activity was observed, resulting in a failure of tumors to progress to malignancy (Kimmelman & White 2017). However, general autophagy also contributes to the tumor phenotype as a result of its role in amino acid recycling and cell-cell interactions in the tumor microenvironment (Kimmelman & White 2017), so it is difficult to assign a specific activity to mitophagy in cancer based on studies that eliminate all autophagy. In this review, we parse out specific functions of mitophagy in cancer with a particular focus on the most thoroughly studied mitophagy regulators in cancer, PINK1 and BNIP3/NIX. We refer the reader to Supplemental Table 1 for an extensive summary of other mitophagy regulators.

## PINK1-DEPENDENT MITOPHAGY IN CANCER

Eliminating depolarized mitochondria through the stabilization of PTEN-induced putative kinase 1 (PINK1) at the outer mitochondrial membrane (OMM) is one of the major mechanisms of mitophagy in the cell (Harper et al. 2018). In healthy mitochondria, PINK1 is imported to the inner mitochondrial membrane (IMM) in a manner dependent on its amino-terminal mitochondrial targeting sequence and on mitochondrial membrane potential (MMP), where it is cleaved in its transmembrane (TM) domain by PINK/PGAM5-associated rhomboid-like (PARL) proteases before being retrotranslocated to the cytosol and degraded by the proteasome according to the N-end rule (Figure 1a) (Harper et al. 2018, Sekine et al. 2019). When mitochondria lose MMP, PINK1 accumulates at the OMM, forms high–molecular weight complexes with the TOM (translocase of outer membrane) complex, and becomes activated through autophosphorylation (Figure 1a) (Harper et al. 2018, Sekine et al. 2019). Increases in PINK1 protein levels can thus be used as a measure of mitophagy induction under certain experimental conditions (Supplemental Table 2). The ATAD3A AAA^+^-ATPase bridges the IMM and OMM to facilitate PINK1 import to the matrix for degradation and to prevent aberrant accumulation of PINK1 ( Jin et al. 2018). Loss or mutation of *ATAD3A* increases mitophagy of both depolarized and healthy mitochondria, an effect that is partially rescued by PINK1 deletion (Jin et al. 2018).

PINK1 is a ubiquitin kinase that phosphorylates key ubiquitinated substrates at the OMM, including the Parkin E3 ubiquitin ligase encoded by *PARK2*. PINK1 phosphorylates Parkin on S65 within its ubiquitin-like domain, removing it from autoinhibition and further recruiting active Parkin to the OMM (Harper et al. 2018). Parkin-catalyzed ubiquitin conjugation of substrates at the OMM, such as VDAC1, MFN2, TBK1 (TANK-binding kinase 1), and MIRO, amplifies the mitochondrial “eat me” signal generated by PINK1 (Figure 1a). These phospho-ubiquitin “eat me” signals recruit various mitophagy cargo receptors (Supplemental Table 1), including p62/SQSTM1, OPTN (Optineurin), NDP52, TAXBP1, and others, that target mitochondria for degradation through interaction with LC3 at the autophagosome (Figure 1a) (Harper et al. 2018, Lazarou et al. 2015).

Parkin and PINK1 were originally identified as encoded by genetic loci (*PARK2* and *PARK6*, respectively) that are mutated in human Parkinson’s disease (PD), implicating mitophagy defects in the etiology of PD (Harper et al. 2018). *PARK2* maps to a common fragile site at human chromosome 6q25–q26 that is frequently deleted in bladder, breast, lung, ovarian, and other cancers (Cesari et al. 2003), while *PARK2* mutations have been linked to glioblastoma, colon cancer, and lung cancer (Veeriah et al. 2010). Similarly, *PARK6* expression (PINK1) is downregulated in glioblastoma and ovarian cancer and occasionally mutated in neuroblastoma (Agnihotri et al. 2016). *Parkin*-null mice are susceptible to spontaneous hepatocellular carcinoma and sensitized to irradiation-induced lymphomagenesis (Fujiwara et al. 2008, Zhang et al. 2011), while loss of either *Parkin* or *Pink1* promoted Kras-driven pancreatic ductal adenocarcinoma (PDAC) (Li et al. 2018).

The tumor-suppressor functions of Parkin in mouse tumor models have been attributed to Parkin’s roles in maintaining mitochondrial function, carrying out efficient oxidative phosphorylation, preventing oxidative stress, and ensuring proper lipid homeostasis as a result of effective clearance of depolarized mitochondria (Kim et al. 2011, Li et al. 2018, Zhang et al. 2011). However, both PINK1 and Parkin also suppress HIF-1α stabilization (Agnihotri et al. 2016, Li et al. 2018, J. Liu et al. 2017), with Parkin interacting directly with HIF-1α and ubiquitinating it on K477 to promote its degradation ( J. Liu et al. 2017). Low Parkin levels correlate with high HIF-1α levels and reduced metastasis-free survival in human breast cancer ( J. Liu et al. 2017). Increased Warburg metabolism observed in Kras-driven PDAC when *Parkin* or *Pink1* was deleted was also associated with increased ROS and HIF-1α levels and rescued by HIF-1α deletion or iron chelation (Li et al. 2018). Thus, control of HIF-1α levels may also contribute to the effect of PINK1 or Parkin loss/inactivation in promoting Warburg metabolism in human cancers. Finally, Parkin’s ubiquitin ligase function in cell cycle–controlled degradation complexes, including the FBX4 Cullin-RING ligase complex that regulates cyclin levels, and its interaction with CDC20 to enforce mitotic checkpoint control and genome stability (Gong et al. 2014, Lee et al. 2015) suggest that Parkin may have additional growth-suppressive functions that are independent of its role in mitophagy.

Despite its prominence in the literature as a regulator of mitophagy, the Parkin E3 ubiquitin ligase is not widely expressed even in healthy cells and tissues, and indeed analysis of Parkin-dependent mitophagy has frequently been performed under conditions where exogenous Parkin is overexpressed (Lazarou et al. 2015, Orvedahl et al. 2011, Sumpter et al. 2016). However, several other E3 ubiquitin ligases have emerged as compensating for Parkin in mitophagy, including MUL1 E3 ubiquitin ligase, which is required for elimination of paternal mitochondria during embryogenesis (Rojansky et al. 2016, Yun et al. 2014). In contrast to Parkin, the ARIH1 E3 ubiquitin ligase is widely expressed in cancer cell lines and, importantly, was shown to be dependent on PINK1 activity for its mitophagy-promoting functions (Villa et al. 2017). ARIH1 is induced by chemotherapeutic agents, such as cisplatin and etoposide, where it has been proposed that ARIH1-dependent mitophagy protects tumor cells from therapy-induced cell death to promote drug resistance (Villa et al. 2017).

## AUTOPHAGY CARGO RECEPTORS IN MITOPHAGY AND CANCER

Autophagy cargo receptors, such as the widely studied p62/SQSTM1, interact with processed LC3 at the autophagosome through a conserved LC3-interacting region (LIR) motif and separately with ubiquitinated chains on the cargo to promote targeted degradation by autophagy (Moscat et al. 2016). For example, p62/SQSTM1 facilitates turnover of mitochondria in a PINK1/Parkin-dependent manner by interacting with ubiquitinated VDAC1 at the OMM following mitochondrial depolarization (Geisler et al. 2010). However, p62/SQSTM1 promotes the autophagic turnover of a wide range of cellular cargoes, and thus p62/SQSTM1 is not a mitophagy-selective adaptor. In addition, p62/SQSTM1 is a multifunctional protein implicated in control of NRF2, mTOR, and other cancer-relevant activities beyond autophagy, and the ubiquitin-binding domain in p62/SQSTM1 is not required for it to promote tumorigenesis (Moscat et al. 2016). Thus, the role of p62/SQSTM1 in cancer cannot be simply attributed to its ability to promote mitophagy.

The OPTN, NDP52, and TAX1BP1 cargo receptors may be more critical for mitophagy than p62/SQSTM1 (Supplemental Table 1) (Heo et al. 2015, Lazarou et al. 2015, Moore & Holzbaur 2016, Wong & Holzbaur 2014). OPTN is inactivated in amyotrophic lateral sclerosis (ALS) and is essential for PINK1/Parkin-dependent mitophagy (Moore & Holzbaur 2016, Wong & Holzbaur 2014). ALS-linked mutations in OPTN prevent its recruitment to mitochondria, suggesting that ALS-associated neuronal defects are due to defective mitophagy (Wong & Holzbaur 2014). TBK1 is a Parkin/PINK1 target and phosphorylates OPTN, promoting the co-recruitment of OPTN and TBK1 together to depolarized mitochondria. Similar to OPTN, mutations in TBK1 that are associated with ALS block its ability to promote mitophagy (Moore & Holzbaur 2016). TBK1 is also activated by the cGAS-STING pathway (Vanpouille-Box et al. 2018), suggesting a link between the sensing of cytosolic DNA, including mtDNA, and induction of mitophagy. OPTN expression is reduced in lung cancer cell lines, and higher OPTN expression is correlated with improved relapse-free survival in lung cancer patients (Liu et al. 2014). Both NDP52 and TAX1BP1 have also been implicated in PINK1-dependent mitophagy, although not as extensively as OPTN (Moore & Holzbaur 2016).

## FUNCTIONS OF BNIP3 AND NIX (BNIP3L) IN CANCER

In addition to removing damaged mitochondria via PINK1-dependent mitophagy, cells also turn over healthy mitochondria as an adaptive response to nutrient stress. BNIP3 and NIX (also known as BNIP3L) are stress-induced mitophagy receptors that interact directly with LC3 to promote the turnover of otherwise healthy mitochondria (Figure 1b). Both BNIP3 and NIX are distantly related to proapoptotic BH3 proteins, although the BH3 domains in BNIP3 and NIX are poorly conserved (2 amino acids out of 11) and are redundant for BNIP3/NIX function (Ney 2015). Moreover, BNIP3 and NIX promote cell survival under various physiological contexts, while loss of BNIP3 and NIX sensitizes to cell death (Glick et al. 2012, Ney 2015).

Transcriptional control of BNIP3 and NIX expression is tightly regulated and responsive to various physiological stresses, including hypoxia, nutrient availability, and DNA damage. Both BNIP3 and NIX are HIF-1 targets, although BNIP3 is more sensitive to oxygen decreases than NIX due to differential dependence on HIF-1 transactivation domains (Bruick 2000, Kasper et al. 2005). BNIP3 and NIX are also p53 transcriptional targets (Fei et al. 2005, Feng et al. 2011), although BNIP3 is repressed by p53 (Feng et al. 2011) while NIX is induced (Fei et al. 2005), suggesting that each may respond differently to p53 activation and DNA damage signaling. In addition, BNIP3 is transcriptionally regulated by FOXO3a during muscle atrophy (Mammucari et al. 2007), PPARα in fasted liver (Lee et al. 2014), RB/E2F during cell cycle control (Tracy et al. 2007), NF-κB during apoptosis (Shaw et al. 2008), and oncogenic Ras (Kalas et al. 2011). Consistent with their expression patterns, BNIP3 and NIX both promote mitophagy in the ischemic heart, while BNIP3 plays a more significant role in atrophying skeletal muscle and fasted liver (Glick et al. 2012, Lee et al. 2011, Mammucari et al. 2007). NIX is required for the differentiation of retinal ganglion cells during embryogenesis when it is induced in mid-gestation by hypoxia, resulting in a glycolytic switch (Esteban-Martinez et al. 2017). This NIX-dependent glycolytic switch is also seen during the polarization of M1 macrophages (Esteban-Martinez et al. 2017). NIX is essential for mitophagy during normal red blood cell differentiation, and loss of NIX results in anemia and splenomegaly (Diwan et al. 2007, Sandoval et al. 2008, Schweers et al. 2007). NIX is dramatically upregulated during erythroblast differentiation where its expression is induced by sphingosine kinase 1 (SPHK1) (Diwan et al. 2007, Yang et al. 2019), which is notable since SPHK1 localizes to the mitochondria in response to mitochondrial stress and is required for UPR^mt^ (Kim & Sieburth 2018), suggesting that NIX expression may be responsive to mitochondrial stress downstream of SPHK1.

BNIP3 and NIX are both tail-anchored proteins that integrate into the OMM via a C-terminal TM domain (Figure 1b) (Novak et al. 2010, Vande Velde et al. 2000). The TM domain of BNIP3 has been crystallized and consists of a glycine zipper that mediates tight homodimerization via hydrogen bonding between polar residues on the inside of the glycine zipper, while hydrophobic residues on the outside of the zipper stabilize the dimer in the OMM’s lipid bilayer (Sulistijo & MacKenzie 2009). Most studies of BNIP3 and NIX focus on how they function in mitophagy as homodimers, but these highly related proteins can also heterodimerize (Ohi et al. 1999), although the functional significance of their heterodimerization for mitophagy remains unclear. The amino termini of BNIP3 and NIX extend into the cytosol, where they interact to promote mitophagy with LC3-related molecules at nascent phagophores via conserved LIRs located at amino acids 15 to 21 in BNIP3 and 43 to 49 in NIX (Hanna et al. 2012, Schwarten et al. 2009). Both BNIP3 and NIX turn over with mitochondria, and thus their continued induction under stress is required to maintain expression.

NIX is ubiquitinated by Parkin and promotes PINK1/Parkin-induced mitophagy (Ding et al. 2010, Gao et al. 2015). However, neither BNIP3 nor NIX requires PINK1 or Parkin to elicit mitophagy (Zhang et al. 2016), and conversely, BNIP3 is not required for PINK1 accumulation or for recruitment of Parkin to the OMM (Zhang et al. 2016). Parkin or PINK1 loss promotes HIF-1 stabilization (Li et al. 2018, J. Liu et al. 2017), suggesting that, as HIF-1-target genes, *BNIP3* and *NIX* may be upregulated by PINK1/Parkin loss, and indeed, BNIP3 can compensate for Parkin loss in mitophagy (Zhang et al. 2016). The functional interaction of PINK1/Parkin and BNIP3/NIX in genetic cancer models has not been examined.

Switching cells from glucose to glutamine/galactose as a source of carbon induced NIX-dependent mitophagy, which required recruitment of the small GTPase Rheb to the OMM, where it interacted directly with both NIX and LC3 in a trimolecular complex to promote mitophagy (Melser et al. 2013). It was proposed that Rheb-induced mitophagy promoted oxidative efficiency under the new growth conditions by eliminating damaged mitochondria (Melser et al. 2013). Canonically, RHEB interacts with mTOR at the lysosome in the presence of amino acids to activate mTOR and promote cell growth (Vyas et al. 2016). However, the noncanonical role for RHEB in promoting mitophagy is reportedly independent of mTOR (Melser et al. 2013). Bnip3 also interacts with Rheb, but this was not linked to mitophagy (Li et al. 2007). Rheb’s GTPase activity was suppressed by its interaction with Bnip3, resulting in reduced mTOR signaling. Conversely, disrupting the Bnip3-Rheb interaction promoted mTOR activity and cell growth, consistent with a growth-suppressive role for BNIP3 (Li et al. 2007). These reports suggest that BNIP3 and NIX differ in their functional interactions with RHEB.

BNIP3 and NIX protein levels are elevated at early stages of various human solid cancers as tumors become hypoxic, including breast cancer (Sowter et al. 2003). The BNIP3 locus at chromosome 10q26.3 is most frequently deleted in metastatic progression of triple-negative breast cancer (TNBC) (Chourasia et al. 2015, Koop et al. 2009). TNBC is the subtype of breast cancer that exhibits the strongest HIF signature and Warburg metabolism (Montagner et al. 2012), and loss of BNIP3 combined with high HIF-1 expression predicted poor metastasis-free survival specifically for TNBC (Chourasia et al. 2015). In an MMTV-PyMT (mouse mammary tumor virus–polyoma middle T antigen) mouse model of breast cancer, loss of Bnip3 promoted tumor growth, resulted in faster progression to invasive carcinoma, reduced latency of lung metastasis, and reduced overall survival (Chourasia et al. 2015). Consistently, Bnip3 knockdown promoted tumor growth and metastasis in an orthotopic model of mammary tumorigenesis (Manka et al. 2005). The increased aggressiveness of mammary tumors in this model lacking Bnip3 (compared to wild-type controls) was linked to reduced mitophagy, dysfunctional mitochondria, increased ROS, and HIF-1 stabilization, with attendant increases in rates of glycolysis and angiogenesis in the tumors (Chourasia et al. 2015). These data suggest that BNIP3 acts as a brake on HIF-1 and that there is selective pressure to inactivate BNIP3 downstream of HIF-1 as tumors progress to becoming invasive (Chourasia et al. 2015). TNBC is the breast cancer subtype with the highest degree of mitochondrial dysfunction, possibly due to defective mitophagy, and this is currently being exploited to develop novel therapeutic approaches (Guha et al. 2018, Lee et al. 2019).

Hypermethylation of the BNIP3 promoter is a common mechanism of BNIP3 inactivation in other human cancers, including gastric, pancreatic, liver, lung, and hematological malignancies (Murai et al. 2005, Okami et al. 2004). Epigenetic silencing of *BNIP3* in human pancreatic cancer was linked to chemoresistance and poor prognosis (Akada et al. 2005, Erkan et al. 2005), consistent with tumor-suppressive functions for BNIP3. Loss of Bnip3 in the KC (*LSL-Kras*^G12D^*;Pdx1-Cre*) mouse model of PDAC resulted in reduced tumor latency and increased progression to metastasis, supporting a tumor-suppressor role for Bnip3 (Macleod et al., unpublished manuscript). Accelerated PDAC in this model was associated with reduced mitophagy in acinar cells and increased acinar-ductal metaplasia at early stages of tumorigenesis. By contrast, loss of NIX in the KPC (*LSL-Kras*^G12D^*;Tp53*^R172H^*;Pdx1-Cre*) model of PDAC delayed tumorigenesis (Humpton et al. 2019) and was associated with reduced mitophagy, increased oxidative metabolism, and attenuated progression from the pancreatic intraepithelial neoplasia stage to PDAC; consistently elevated NIX expression in a cohort of PDAC patients was linked to worse prognosis (Humpton et al. 2019). These findings point to divergent roles for BNIP3 and NIX in pancreatic cancer, with BNIP3 acting as a tumor suppressor and NIX as a tumor promoter. These differences may be explained by the expression of BNIP3 and NIX at different stages of disease or in different cell types (acinar versus ductal) and require further investigation.

## MITOCHONDRIAL FISSION PROMOTES MITOPHAGY OF DEPOLARIZED MITOCHONDRIA

During starvation periods, healthy mitochondria are protected from turnover by OPA1-dependent fusion accompanied by inhibition of fission at functional mitochondria (Gomes et al. 2011, Rambold et al. 2011). By contrast, depolarized mitochondria fail to fuse, undergo fragmentation, and are preferentially targeted for degradation (Gomes et al. 2011, Rambold et al. 2011). In addition to its critical role in mitochondrial fission (Mishra & Chan 2014), DRP1 is involved in selecting dysfunctional regions of the mitochondrial network for mitophagy (Cho et al. 2019). DRP1 is activated by loss of MMP (ΔΨ_mt_) and is recruited to the OMM via MFF and FIS1 receptors (Mishra & Chan 2014). FIS1 is also required for PINK1 recruitment to mitochondria in leukemia stem cells and for activation of mitophagy in response to AML (acute myeloid leukemia) therapy (Pei et al. 2018). At this time, it is not clear whether FIS1 has additional functions in mitochondria that contribute to its ability to promote mitophagy, such as integrating fission activity with the autophagy machinery.

At the OMM, DRP1 also interacts with ZIP1 to regulate MMP via altered mitochondrial uptake of Zn^2+^ through the mitochondrial calcium uniporter (Cho et al. 2019). The DRP1-ZIP1 interaction takes place at sites of mitochondrial fission, although GTPase-deficient DRP1 can still interact with ZIP1 to promote ΔΨ_mt_, indicating that DRP1 can regulate MMP independent of its role in fission (Cho et al. 2019). The DRP1-ZIP1 interaction was required for recruitment of GFP (green fluorescent protein)-Parkin to mitochondria in response to hyperglycemia, and chelating Zn^2+^ or blocking the DRP1-ZIP1 interaction impeded ΔΨ_mt_, GFP-Parkin recruitment, and mitophagy (Cho et al. 2019). This caused short mitochondria with reduced MMP to accumulate, as damaged mitochondria could not be turned over by mitophagy. Mitochondrial recruitment of ARIH1 was also dependent on DRP1-ZIP1. These results explain how fission is coordinated with mitophagy to promote selective turnover of regions of the mitochondrial network that are depolarized or damaged.

RAS transformation of fibroblasts induces ΔΨ_mt_, increased mitochondrial ROS, and DRP1-dependent mitochondrial fission (Serasinghe et al. 2015), and DRP1 phosphorylation on S616 by ERK2 is required for RAS transformation (Kashatus et al. 2015). RAS also induces expression of BNIP3 and NIX (Humpton et al. 2019, Kalas et al. 2011), but it is not yet clear if DRP1-induced fission is required for RAS transformation to promote mitophagy or simply to promote fission.

In addition to its role in selecting depolarized mitochondria for turnover, mitochondrial fission has also been postulated to be required for mitophagy due to the limiting size constraints on autophagosomal cargo. However, turnover of the endoplasmic reticulum (ER) by ER-phagy occurs in a piecemeal manner, thereby raising the probability of piecemeal mitophagy. MTX1 was identified as an OMM protein that functions as an autophagy receptor in piecemeal mi-tophagy that interacts with LC3C, does not require fission or PINK1/Parkin to function, and plays a housekeeping function in cells (Le Guerroue et al. 2017). Thus, it remains to be determined to what extent different mitophagy pathways rely on DRP1 or fission to promote mitochondrial turnover.

## SENSING MITOCHONDRIAL DYSFUNCTION TO ACTIVATE MITOPHAGY

The stabilization of PINK1 at the OMM in response to ΔΨ_mt_ explains how one type of mitochondrial dysfunction induces mitophagy. However, signaling pathways that induce mitophagy in response to other mitochondrial stresses, such as inhibition of respiration, imbalanced nuclear-to-mitochondrial protein levels, mtDNA mutation, and elevated mitochondrial ROS production, have not been fully elucidated. ATF4 is induced by mitochondrial stress (Quiros et al. 2017), including respiratory chain inhibition and the UPR^mt^, but a link between ATF4 and mitophagy has not been made. BNIP3 and NIX are transcriptionally induced by factors that are sensitive to ROS (HIF-1, FOXOs, p53, and NF-κB) linking BNIP3/NIX-dependent mitophagy to elevated mitochondrial ROS. However, there remains much to be determined about how mitochondrial dysfunction induces mitophagy.

Reduced respiration arising from mtDNA mutation or exposure to respiratory chain inhibitors, such as metformin, impairs the efficiency of ATP synthesis and activates AMPK (Herzig & Shaw 2018, Martinez-Reyes et al. 2016). AMPK activation was associated with induction of mitophagy arising from extended mitotic arrest (Domenech et al. 2015) where AMPK phosphorylated the glycolysis regulator PFKFB3 to promote glycolysis and survival (Domenech et al. 2015). AMPK also induces general autophagy via phosphorylation and activation of ULK1 (Egan et al. 2011), and AMPK phosphorylates both MFF and FIS1 (Pei et al. 2018, Toyama et al. 2016), which are mitochondrial receptors for DRP1, and thus could contribute to mitophagy induction indirectly. Loss of LKB1, the regulatory kinase upstream of AMPK (Herzig & Shaw 2018), leads to mitochondrial dysfunction, including lower oxygen consumption, altered expression of electron transport chain components, increased ROS production, lower ATP production, and altered lipid and amino acid metabolism (Gurumurthy et al. 2010, Kottakis et al. 2016, Masand et al. 2018, Nakada et al. 2010). However, it is not fully resolved to what extent this was due to defective AMPK activation or the result of defective mitophagy (He et al. 2017, Yang et al. 2017); this is another area requiring further investigation.

Many of the stresses arising from defective mitophagy (ROS, NAD^+^ depletion, AMPK activation, and cytosolic mtDNA) interact in feedback loops to activate mitophagy pathways, resulting in amplification of mitochondrial damage when mitophagy is inactivated (Figure 2). For example, failure to remove dysfunctional mitochondria by mitophagy leads to high ROS and cell death, including ferroptosis, a necrosis-like cell death that results from redox stress and lipid peroxides (Gao et al. 2019). Cysteine depletion induces mitochondrial lipid ROS, mitochondrial fragmentation, depolarization, and cristae disruption prior to ferroptosis. Depletion of mitochondria through overexpression of Parkin in combination with uncoupling agent CCCP (carbonylcyanide-3-chlorophenylhydrazone) protected against ferroptosis (Gao et al. 2019).

## MITOCHONDRIAL DYSFUNCTION IN CELLULAR SENESCENCE

Age-related nonalcoholic fatty liver disease has been linked to dysfunctional mitochondria in senescent hepatocytes that were unable to oxidize fats efficiently, and lipid accumulation in ageing liver was prevented by eliminating senescent hepatocytes (Ogrodnik et al. 2017). Inhibition of PGC1β-dependent mitochondrial biogenesis reduced mitochondrial mass and attenuated age-related senescence in mouse liver, consistent with signals from dysfunctional mitochondria promoting senescence (Correia-Melo et al. 2016). Indeed, mitochondrial dysfunction, including decreased oxidative phosphorylation and elevated ROS, is a key feature of senescent cells that is associated with reduced mitophagy (Wiley et al. 2016). Promoting mitophagy by overexpressing Parkin in the presence of CCCP to uncouple mitochondrial respiration inhibited DNA damage–induced replicative senescence, indicating that dysfunctional mitochondria are required for senescence and that mitophagy limits cellular senescence (Correia-Melo et al. 2016).

Artificially inducing mitochondrial dysfunction by treating cells with respiratory chain inhibitors, depleting mtDNA, or knocking down mitochondrial Sirtuins (Sirt3 or Sirt5) induced cellular senescence in primary human fibroblasts and was termed MiDAS (mitochondrial dysfunction–associated senescence) (Wiley et al. 2016). Pyruvate blocked MiDAS (Wiley et al. 2016), suggesting that NAD^+^ depletion arising from complex I inhibition stimulates cellular senescence (Hosios & Vander Heiden 2018), possibly as a result of Sirtuin inactivation (Vyas et al. 2016). Mitochondrial dysfunction also induced AMPK, and dominant negative AMPK conferred resistance to MiDAS (Wiley et al. 2016), but whether this was associated with altered rates of mitophagy was not examined. AMPK activation by mitochondrial dysfunction did contribute to a p53- and p16-dependent growth arrest during MiDAS. Interestingly, the senescence-associated secretory phenotype associated with MiDAS was distinct from that induced by DNA damage (Wiley et al. 2016). This raises the question of whether mtDNA is released during MiDAS to activate the NLRP3 inflammasome or cGAS-STING pathway as a means of modulating cytokine production, as discussed below. In summary, cellular senescence is linked to mitochondrial dys-function, possibly arising from defects in mitophagy in a variety of physiological settings, although the expression and activity of specific mitophagy receptors during cellular senescence remain to be determined.

## MITOPHAGY IN TISSUE STEM CELLS AND CANCER STEM CELLS

The balance between glycolysis and oxidative metabolism has been reported in various systems to determine rates of stem cell quiescence versus differentiation (Ito & Suda 2014). Reduced respiratory capacity is required for self-renewal of hematopoietic stem cells (HSCs) and is achieved through increased mitophagy of respiring mitochondria to limit ATP generation and maintain stem cells in a slow-cycling state (Ho et al. 2017, Ito et al. 2016, Vannini et al. 2016). Activating autophagy in older HSCs promoted quiescence and protected against replication stress and ageing (Ho et al. 2017). Mitophagy also plays a role in the preferential segregation of younger, more functional mitochondria to daughter stem cells compared to nonstem cells (Katajisto et al. 2015). However, recent work has suggested that mitochondrial mass in stem cells is underestimated due to experimental overreliance on MitoTracker^®^ and other mitochondrial dyes that are pumped out of stem cells more efficiently than nonstem cells (de Almeida et al. 2017). This study did not dispute that HSCs have a lower respiratory capacity than nonstem cells but suggested that this was not explained by altered mitochondrial mass. However, this study relied on measurement of mtDNA and effects of depolarization with CCCP that are also subject to criticism (Supplemental Table 2). A more recent study employing the genetic tools Mito-EGFP (enhanced GFP) and Mito-QC (quality control) to measure mitochondrial mass and mitophagy, respectively, as opposed to mitochondrial dyes, has confirmed previous data showing that HSCs do indeed have fewer mitochondria than nonstem cells and that mitophagy is more readily induced in HSCs than non-HSCs (Vannini et al. 2019). These studies emphasize the importance of employing relevant techniques and not overinterpreting mitochondrial mass data without directly parsing what is due to mitophagy versus biogenesis (Supplemental Table 2).

Cancer stem cells (CSCs) contribute to the most insidious aspects of human cancer, including the ability to promote distant metastases, therapy resistance, tumor dormancy, and minimal residual disease (Shibue & Weinberg 2017). Like tissue stem cells, CSCs are dependent on general autophagy for many aspects of their phenotype (Smith & Macleod 2019), and more recently, CSCs have been reported to exploit mitophagy to maintain their self-renewal properties (K. Liu et al. 2017, Pei et al. 2018, Whelan et al. 2017). Parkin-dependent mitophagy in esophageal squamous cell carcinoma cells was essential to prevent cell death during epithelial-to-mesenchymal transition and for the expression of the stem cell marker CD44 (Whelan et al. 2017). Mitophagy was also required to maintain hepatic CSCs by eliminating mitochondrial p53 (K. Liu et al. 2017). Mi-tophagy inhibition, PINK1 activation, and PINK1-dependent phosphorylation of p53 promoted the nuclear translocation of p53, where it antagonized expression of the stem cell factor NANOG (K. Liu et al. 2017). *CD44* is a p53-target gene, suggesting that mitophagy-dependent regulation of CD44 levels may be p53 dependent (Smith & Macleod 2019). Turnover of transcription factors that get sequestered at mitochondria could be a more broadly acting mechanism to explain how mitophagy controls cell fate and stress responses (Smith & Macleod 2019).

## MITOPHAGY ATTENUATES ACTIVATION OF cGAS-STING AND THE NLRP3 INFLAMMASOME

Mitochondrial dysfunction can result in release of mtDNA to the cytoplasm and activation of both the cGAS-STING pathway and the NLRP3 inflammasome to promote antitumor immunity (Figure 2) (Chen et al. 2016, Vanpouille-Box et al. 2018, Zhou et al. 2011). Activation of the cGAS-STING pathway by cytosolic mtDNA induces a type I interferon response mediated via activation of TBK1 and IRF3 (Chen et al. 2016). Activation of TBK1 also promotes mitophagy through recruitment of cargo receptors, such as OPTN, while STING trafficking itself stimulates autophagy by promoting autophagosome biogenesis at the ER-Golgi junction (Gui et al. 2019). Thus, TBK1 activation provides a key link between dysfunctional mitochondria sensed via cytosolic mtDNA and mitophagy activation.

Oxidized mtDNA, or newly synthesized mtDNA that has yet to be packaged into TFAM-positive nucleoids, is released from damaged mitochondria and binds directly to the NLRP3 inflammasome to induce procaspase-1 activation and IL-1β release (Shimada et al. 2012, Vanpouille-Box et al. 2018, Zhong et al. 2018). Caspase-1 activation at the mitochondria induces further mitochondrial damage, including ΔΨ_mt_, ROS generation, and mtDNA release that amplify NLRP3 activation in a feedback loop. NLRP3 inflammasome activation induces Parkin-dependent mitophagy (Zhong et al. 2016) although Parkin is cleaved by caspase-1 to limit mi-tophagy with the resultant excess inflammation leading to pyroptosis, again allowing caspase-1 to amplify its own activation (Yu et al. 2014).

In summary, by eliminating damaged mitochondria, mitophagy likely limits mtDNA release and activation of cGAS-STING or the NLRP3 inflammasome in a feedback loop (Chen et al. 2016, Vanpouille-Box et al. 2018, Yu et al. 2014, Zhou et al. 2011). Recent data suggest that the cGAS-STING and NLRP3 pathways communicate with each other via NLRP3 being activated by STING’s effects on the release of potassium from the lysosome (Vanpouille-Box et al. 2018).

## MITOPHAGY IN THE RESPONSE TO DNA DAMAGE AND NAD^+^ DEPLETION

Boosting NAD^+^ levels in cells and tissues of mice by feeding them nicotinamide riboside (NR) promoted long-term hematopoiesis that depended upon the induction of mitophagy in long-term repopulating HSCs (Vannini et al. 2019). NAD^+^ levels in cells are heavily influenced by mitochondrial function and by complex I activity in particular (Figure 2). Inhibition of complex I with metformin or other drugs makes tumor cells more dependent on pyruvate and lactate dehydrogenase activity for amino acid biosynthesis and survival (Hosios & Vander Heiden 2018). DNA damage also depletes cellular NAD^+^ levels due to elevated poly(ADP-ribose) polymerase (PARP) activity that consumes NAD^+^ to catalyze covalent linkage of PARP groups to proteins (Gupte et al. 2017). As a result, NAD^+^-dependent Sirtuins are inhibited by both mitochondrial dysfunction and DNA damage (Figure 2) (Fang et al. 2014, Gupte et al. 2017). Sirtuins modulate many growth-regulatory processes in the cell (Vyas et al. 2016), including the NAD^+^-dependent deacetylation of PGC1α, which is required for mitochondrial biogenesis, fatty acid oxidation (Gerhart-Hines et al. 2011), and the activation of FOXO3a via deacetylation, which promotes the expression of autophagy genes, including *BNIP3* (Mammucari et al. 2007). This suggests that Sirtuins control mitochondrial mass and function. The simultaneous induction of mitochondrial biogenesis and mitophagy by Sirtuins supports the hypothesis that coordinated induction of both processes allows mitochondria to be more efficiently reprogrammed in response to nutrient stress (Ploumi et al. 2017).

Both xeroderma pigmentosum (XP) and ataxia telangiectasia (AT) are cancer predisposition syndromes in which faulty DNA repair plays a major causative role in disease. However, defective mitophagy arising from NAD^+^ exhaustion and reduced Sirtuin activity also contributes to disease etiology (Fang et al. 2014, 2016). In particular, neurodegeneration in XPA individuals is attributed to reduced mitophagy and is rescued by PARP inhibition (Fang et al. 2014). Similarly, loss of ATM kinase caused ΔΨ_mt_, increased mitochondrial ROS (Valentin-Vega et al. 2012), and reduced mitophagy (Fang et al. 2016). Replenishing NAD^+^ levels and activating SIRT1 in ATM-deficient neurons induced mitophagy, inhibited cell death, and promoted neuronal differentiation (Fang et al. 2016).

In *atm-1* worms (*Caenorhabditis elegans*), replenishing NAD^+^ levels with NR promoted mitophagy through increased levels of *dct-1* in a manner dependent on *sirt2* and on the upstream regulator of *dct-1*, *daf-16* (Fang et al. 2016). The gene *dct-1* is the *C. elegans* homolog of *BNIP3*/*NIX* that limits ageing in worms by promoting mitophagy (Palikaras et al. 2015). It is not known whether BNIP3 or NIX plays a similar role in mammalian systems by promoting mitochondrial function downstream of Sirtuins or whether BNIP3 or NIX is reduced in XP or AT, but these are logical future avenues of investigation.

Fanconi anemia (FA) is a different cancer predisposition syndrome linked to defective DNA damage sensing and repair, and like XP and AT, mitophagy is defective in FA (Supplemental Table 1) (Sumpter et al. 2016). FANC-C colocalizes with Parkin at mitochondria in response to depolarization, and FANC-C deficiency caused the accumulation of dysfunctional mitochondria and heightened inflammasome activation (Sumpter et al. 2016), suggesting a direct role for FANC-C in mitophagy.

Overall, defects in mitophagy appear to be a common feature of a variety of cancer predisposition syndromes arising from DNA repair defects, which suggests that treatments to increase mitophagy could benefit patient outcomes. This work also raises the question of whether DNA damage induced by genotoxic agents used in conventional cancer therapy also inhibits mitophagy to cause mitochondrial dysfunction, and if so, does it do so by similar mechanisms? There is selection for epigenetic silencing of BNIP3 in drug-resistant PDAC, and re-expression of BNIP3 promotes drug sensitivity (Akada et al. 2005, Erkan et al. 2005). Currently, general autophagy inhibitors are being proposed as adjuvant therapy in cancer treatments, but if these drugs inhibit mitophagy as would be expected, will this lead to drug resistance by other means?

In conclusion, mitophagy is emerging as a selective form of autophagy that exerts far-reaching effects on cell growth and cell fate, including on cellular senescence, stemness, cellular metabolism, various stress responses including inflammatory responses, and cell viability (Figure 3). Ongoing research may soon allow us to determine whether mitophagy represents a valid therapeutic target separate from efforts to target general autophagy.

## Supplementary Material

1

## Figures and Tables

**Figure 1:**
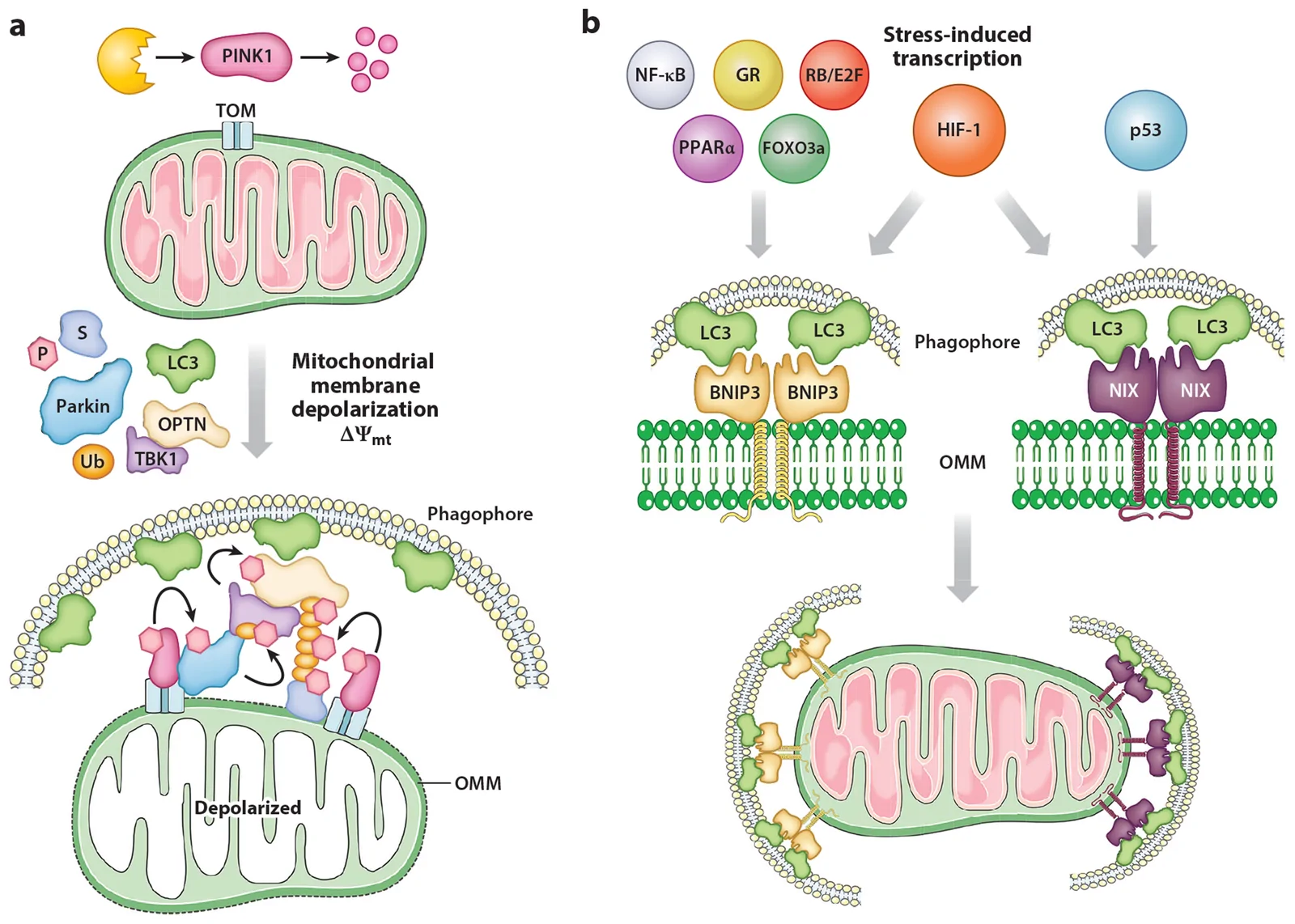
PINK1-dependent (*a*) and BNIP3/NIX-dependent (*b*) mitophagy. (*a*) Proteolytic cleavage of PINK1 is dependent on mitochondrial membrane depolarization (ΔΨ _mt_), and thus stresses that induce ΔΨ_mt_ cause PINK1 kinase to accumulate at the outer mitochondrial membrane (OMM) in a complex with translocase of outer membrane (TOM). PINK1 functions as a ubiquitin kinase that phosphorylates ubiquitin and Parkin (within its ubiquitin-like domain), resulting in recruitment of Parkin ubiquitin ligase activity at the OMM, where it ubiquitinates other substrates (S), such as VDAC1 and MFN2. Interaction of phospho-ubiquitinated mitochondrial targets with processed LC3 requires cargo receptor molecules that simultaneously bind ubiquitin and LC3, including OPTN, which is recruited to mitochondria with TBK1. (*b*) BNIP3 and NIX are stress-induced mitochondrial receptor proteins induced at a transcriptional level by HIF-1 in response to hypoxia. BNIP3 is also transcriptionally induced by PPARα, FOXO3a, RB/E2F, the glucocorticoid receptor (GR), and NF-κB in response to other stresses. Both BNIP3 and NIX interact directly with LC3 through conserved LC3-interacting region motifs in their amino termini to promote targeting of mitochondria to growing phagophore membranes and mitochondrial turnover by autophagy. Figure elements adapted from Servier Medical Art (https://smart.servier.com), under license CC-BY 3.0.

**Figure 2:**
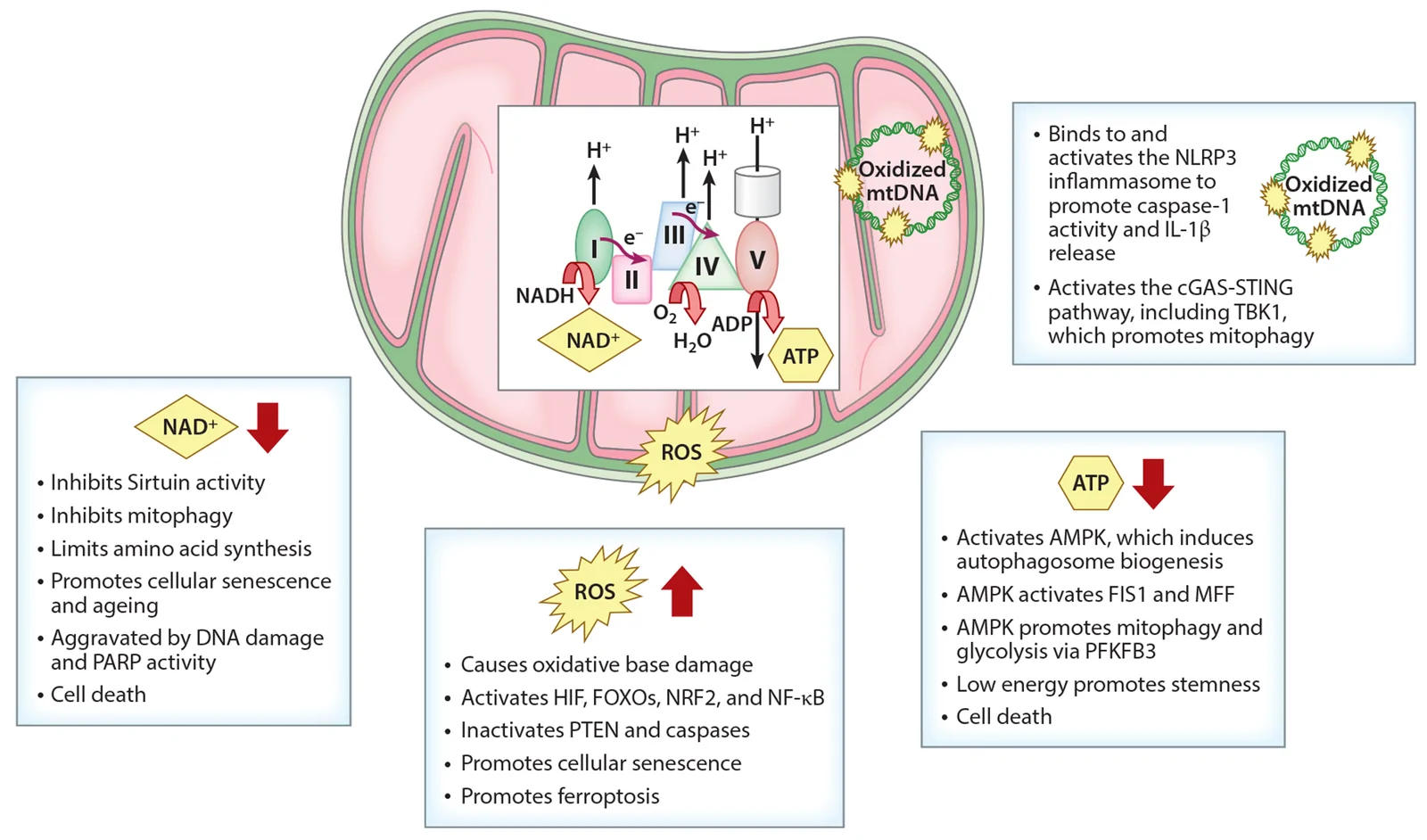
Consequences of mitochondrial dysfunction linked to defective mitophagy. Inhibition of mitophagy causes the accumulation of dysfunctional mitochondria that have membrane depolarization, attenuated capacity to generate NAD^+^ and ATP through oxidative phosphorylation, elevated reactive oxygen species (ROS) production, and release of mitochondrial DNA (mtDNA) to the cytosol, which results in activation of inflammatory responses and ultimately cell death if mitochondrial integrity is not restored. Many of the stresses arising from defective mitophagy (ROS, NAD^+^ depletion, AMPK activation, and cytosolic mtDNA) also themselves activate mitophagy, leading to a vicious cycle of mitochondrial damage when mitophagy is inactivated. It is unclear to what extent other mitochondrial stress responses, including the mitochondrial unfolded protein response, mitochondrial fusion, and mitochondrial biogenesis, can compensate for defective mitophagy. Figure elements adapted from Servier Medical Art (https://smart.servier.com), under license CC-BY 3.0.

**Figure 3:**
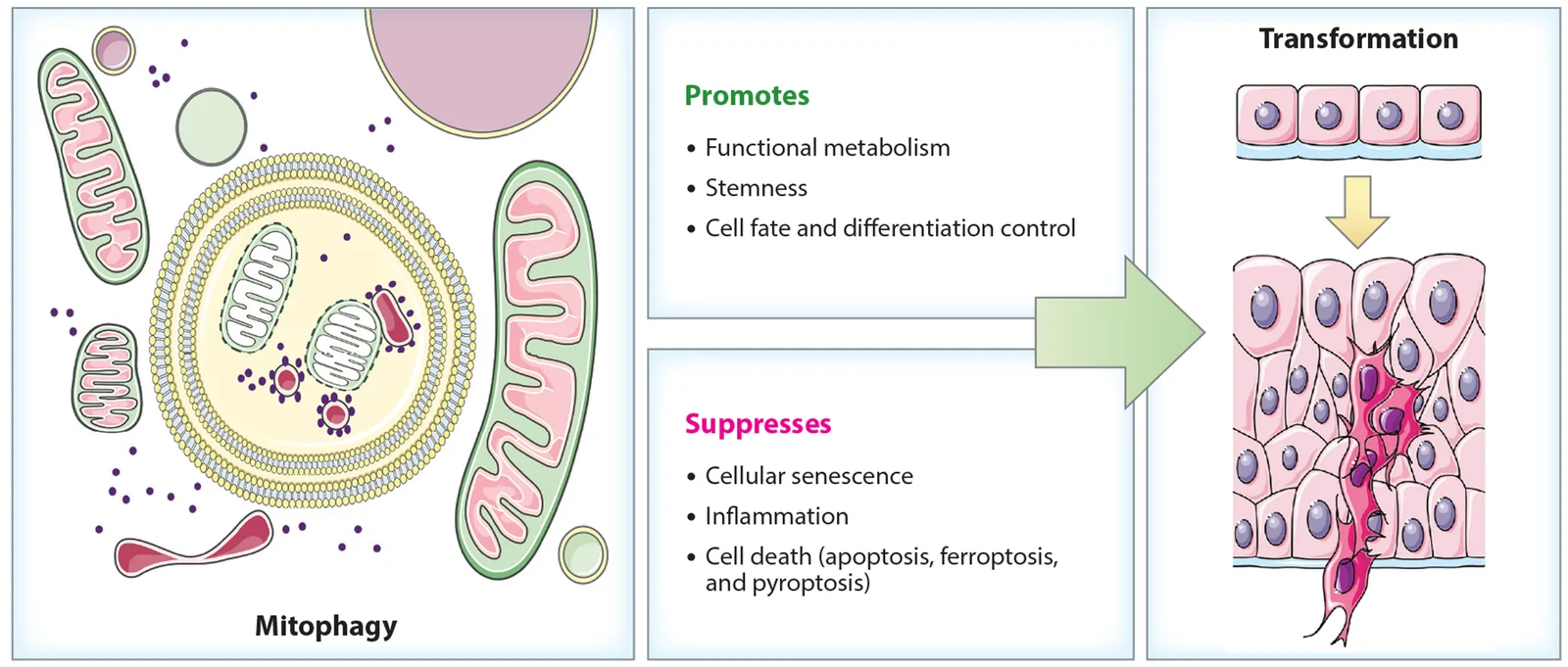
The role of mitophagy in cellular function and transformation. Mitophagy promotes key aspects of mitochondrial metabolism that sustain amino acid, lipid, and nucleotide biosynthesis; supports the stem cell phenotype and different types of differentiation by determining rates of glycolysis; modulates cell fate and differentiation via mitochondrial reprogramming, cellular remodeling, turnover of fate-determining transcription factors sequestered at the mitochondria; limits cellular senescence and alters the secretome of senescent cells; attenuates inflammation by preventing inflammasome and cGAS-STING activation; and reduces cell death by promoting mitochondrial function, including preventing apoptosis, ferroptosis, and pyroptosis. How these cellular housekeeping functions of mitophagy modulate cellular transformation is an area of ongoing investigation. Figure elements adapted from Servier Medical Art (https://smart.servier.com), under license CC-BY 3.0.

## SUMMARY POINTS

1. Mitophagy plays a housekeeping role in normal cells by eliminating dysfunctional or excess mitochondria, ensuring that reactive oxygen species (ROS), cytosolic mitochondrial DNA, and metabolic disequilibrium are limited while sustaining NAD^+^ and ATP levels and the production of key mitochondrial metabolites required for nucleotides, amino acids, and lipids.
2. There are multiple mitophagy pathways in the cell, including PINK1-dependent mitophagy and BNIP3/NIX-dependent mitophagy; readers are referred to Supplemental Table 1 for an extensive list of mitophagy modulators.
3. PINK1-dependent mitophagy eliminates depolarized mitochondria in concert with different E3 ubiquitin ligases, including Parkin, ARIH1, MUL1, and others.
4. BNIP3 and NIX are induced by hypoxia, nutrient deprivation, and other stresses, and interact directly with LC3 to promote mitophagy; BNIP3 and NIX are functionally homologous but their expression in different tissues and stages of tumorigenesis may contribute to distinct roles in development and cancer.
5. DRP1 and mitochondrial fission proteins, including FIS1 and MFF, promote mitophagy by both identifying dysfunctional regions of the mitochondrial network and reducing the size of mitochondria, allowing them to be more readily engulfed by nascent phagophores.
6. Mitochondrial mass in cells is determined by the balance between rates of mitophagy (reducing mass) and mitochondrial biogenesis (increasing mass). Thus, measurement of mitochondrial mass does not equate to measurement of mitophagy; readers are referred to Supplemental Table 2 for more discussion of how to measure mitophagy robustly.
7. Mitophagy limits cellular senescence, promotes stemness, reduces inflammasome and cGAS-STING activation, and prevents cell death, including ferroptosis.
8. The role of mitophagy in cancer is not yet fully understood; based on their expression in human cancer and effects of their deletion in mouse tumor models, PINK1/Parkin and BNIP3 appear to function as suppressors of tumor growth while, intriguingly, NIX may promote tumorigenesis.

## FUTURE ISSUES

1. How mitochondrial dysfunction engages signaling pathways to induce mitophagy is a key area requiring further investigation. Currently, we understand how mitochondrial depolarization and mitochondrial ROS (indirectly) and AMPK activation (indirectly) induce mitophagy, but are there other signaling pathways activated by mitochondrial dysfunction that induce mitophagy? For example, the role of mitochondrial lipid signaling pathways in mitophagy, such as those activating sphingosine kinase activity, requires further analysis; mitochondrial sequestration of transcription factors (such as p53), released or alternatively turned over in response to mitochondrial stress, may explain how mitophagy is activated or how mitophagy alters cell fate. Do specific components of the mitochondrial unfolded protein response engage with the mitophagy machinery?
2. Much remains to be determined about how the different mitophagy pathways interact with each other. For example, BNIP3 and NIX are both induced by hypoxia to promote mitophagy, but so is FUNDC1, and it is not clear how or if these mitophagy modulators interact. Conflicting data on how PINK1-dependent mitophagy interacts with BNIP3/NIX-dependent mitophagy need to be clarified through robust genetic and in vivo analyses.
3. PINK1 and Parkin play a role in cell cycle and HIF-1 stabilization alongside their function in mitophagy. There is a need to separate the different functions of PINK1/Parkin to determine how PINK1-dependent mitophagy specifically contributes to tumor phenotypes observed in humans and mice when PINK1 or Parkin is deleted or mutated.
4. BNIP3 and NIX are upregulated following matrix detachment, and further investigation is needed into the role of BNIP3/NIX-dependent mitophagy in metastasis that arises from the survival of detached cells.
5. Areas of ongoing research include how mitophagy is coordinated with mitochondrial biogenesis to achieve wholesale mitochondrial replacement as an adaptation to certain stresses in normal tissues, and how this is deregulated in cancer, seemingly to promote biogenesis at the expense of mitophagy.
6. The role of mitophagy in cellular reprogramming is an area of expanding interest, including dedifferentiation/redifferentiation events that occur during oncogenic processes such as metaplasia through the elimination of mitochondria to modulate energy availability, differentiation-promoting metabolites, the sequestration of key transcription factors, etc.
7. Cancer cachexia, in which loss of muscle and fat mass in cancer patients reduces body weight and the ability of the patient to tolerate treatment regimens and survive, has been linked to mitochondrial dysfunction and elevated mitophagy. It remains to be determined to what extent mitophagy contributes to how tumor growth promotes atrophy of muscle and adipose tissues in cachexia.
