# Supplementary material for: Mitophagy and Mitochondrial Dysfunction in Cancer
Source: Annu Rev Cancer Biol. Author manuscript; Available in PMC 2026 Oct 1. (PMC13626216; doi:10.1146/annurev-cancerbio-030419-033405)
Supplement: Supplementary file 1 [file NIHMS2210122-supplement-1.pdf]

Downloaded from www.annualreviews.org. University of Chicago (ar-129425) IP: 205.208.121.32 On: Tue, 22 Sept 2026 21:37:54

Supplemental Table 1. Summary of key regulators of mitophagy.

Gene/Protein	Mitophagy Function	Cancer/disease relevance	Reference
PINK1-dependent mitophagy			
PARK2/Parkin	E3 ubiquitin ligase, activated by PINK1 through phosphorylation on S65 in its UBL domain causing it to localize to the OMM; conjugates ubiquitin chains to numerous OMM proteins, including Mfn2, VDAC1, TBK1; phospho-Ub chains are bound by autophagy cargo receptors, like OPTN, NDP52, TAXBP1. Antagonized by USP30 and other mitochondrial de-ubiquitinases.	PARK2 inactivating mutations linked to Parkinson's Disease; deleted in human ovarian, breast, lung and bladder cancers; inactivating mutations found in glioblastoma and other cancers; Parkin null mice develop spontaneous liver tumors, are sensitized to radiation-induced lymphoma, loss of Parkin promotes KRas-driven PDAC.	(Lazarou et al 2015) (Heo et al 2015, Veeriah et al 2010);(Li et al 2018)
PARK6/PINK1	Serine/threonine ubiquitin kinase that undergoes voltage-dependent degradation at the mitochondria; stabilized at the OMM by $\Delta\Psi_{mt}$; its import is promoted by Atad3a that prevents its aberrant accumulation; PINK1 phosphorylates ubiquitin chains, and Parkin to derepress its auto-inhibitory activity leading to Parkin recruitment to and activity at the OMM. Functions in concert with other E3 Ub ligases at the mitochondria, including ARIH1, to promote mitophagy.	PARK6 loss linked to Parkinson's disease. Reduced PARK6 expression detected in glioblastoma and ovarian cancer; *PARK6* mutated in rare cases of neuroblastoma; loss of Pink1 promotes KRas-driven PDAC.	(Heo et al 2015) (Agnihotri et al 2016) (Li et al 2018)
PARK8/LRRK2	*PARK8* is a novel PD predisposition locus that encodes LRRK2. LRRK2 has specific kinase activity for Rab GTPases involved in cellular trafficking (Rab1b, Rab8a, Rab10); mutant LRRK2 (G2019S) has increased kinase activity for these substrates. LRRK2 also promotes the degradation of Miro thereby limiting mitochondrial motility and promoting mitochondrial sequestration and mitophagy.	Mutated (*eg.* G2019S) in Parkinson's Disease	(Hsieh et al 2016)
Atad3a	Interacts with TOM40 at the OMM and TIM23 at the IMM and promotes PINK1 import into the mitochondrial matrix for degradation.	Mutant Atad3a (R528W) associated with increased mitophagy in human fibroblasts. Loss of Atad3a causes aberrant accumulation of PINK1.	(Jin et al 2018)
ARIH1	A mitochondrial E3 ubiquitin ligase that can substitute for Parkin, is	Expressed in breast and lung cancer cell lines; protects	(Villa et al 2017)

Downloaded from www.annualreviews.org. University of Chicago (ar-129425) IP: 205.208.121.32 On: Tue, 22 Sept 2026 21:37:54

	dependent on PINK1, DRP1 and ZIP1 for mitophagy.	against cell death induced by genotoxic agents.	
MARCH5 (also known as MITOL)	A mitochondrial RING-finger E3 ligase that plays a role in mitochondrial dynamics via ubiquitination of DRP1 and Mfn1/Mfn2. Recently shown to ubiquitinate and promote degradation of the FUNDC1 mitophagy receptor to attenuate hypoxia-induced mitophagy.	Not known.	(Chen et al 2017b, Sugiura et al 2013)
Mul1	Mitochondrial E3 ubiquitin ligase that can compensate for loss of Parkin in mitophagy.	Not known.	(Yun et al 2014) (Rojansky et al 2016)
Mitophagy cargo receptors			
NDP52 (Nuclear Dot Protein 52); also known as CALCOCO2 (calcium-binding and coiled-coil domain-containing protein-2).	NDP52 interacts with LC3 and is required for Parkin-dependent mitophagy; recruits LC3, DFCP1 and WIPI1 to focal regions of the OMM.	Not known.	(Lazarou et al 2015);(Heo et al 2015);
NBR1 (Near BRCA1)	NBR1 acts as an autophagy cargo receptor protein by binding directly to LC3 through a conserved LIR and to ubiquitinated cargo proteins. Role in mitophagy not established but cannot be excluded. Promotes cell motility by facilitating focal adhesion turnover by autophagy.	Deleted along with BRCA1 in some hereditary breast cancers.	(Kenific et al 2016)
OPTN/Optineurin	OPTN is an autophagy cargo receptor that gets recruited to depolarized mitochondria in a Parkin-dependent manner; similar to other cargo adaptors binds LC3 and ubiquitin. Requires to be phosphorylated by TBK-1 to bind ubiquitin and conversely, OPTN binding to ubiquitin promotes TBK-1 activity in a feedforward loop.	Mutated in ALS. Down-regulated in lung cancer cell lines. Low expression correlates with poor prognosis in human lung cancer.	(Harper et al 2018, Lazarou et al 2015, Wong & Holzbaur 2014)
SQSTM1/p62	Multi-functional protein that acts as a signaling hub integrating numerous stress responses; relevant function here is as a cargo receptor that can promote mitophagy by binding to ubiquitinated proteins at the OMM	Amplified at chr.5 in human kidney cancer, accumulates in pancreatitis and non-alcoholic steatohepatitis, over-expressed in autophagy deficient cancers in mice, required for Ras-mediated	(Li et al 2013, Moscat et al 2016)

Downloaded from www.annualreviews.org. University of Chicago (ar-129425) IP: 205.208.121.32 On: Tue, 22 Sept 2026 21:37:54

	and to processed LC3. Function as a cargo receptor is not specific to mitophagy. Plays other non-autophagy roles in cancer, including modulating NRF2 activation.	transformation in mouse models. Role in cancer is context specific – appears to function distinctly in the tumor from in the tumor microenvironment.	
TAX1BP1 (Tax1 Binding protein)	Like OPTN1 and NDP52, TAX1BP1 is an autophagy cargo receptor that restores mitophagy in cargo receptor deficient cells exposed to CCCP. However, it does so less well than either OPTN or NDP52.	Not known.	(Lazarou et al 2015)
BNIP3/NIX-dependent mitophagy			
BNIP3	Stress-induced OMM mitochondrial protein that binds directly to LC3; induced by hypoxia, nutrient deprivation, oncogenic Ras, FoxO3A, E2F, NF-kB, glucocorticoid receptor; repressed by pRB and p53; required for glucagon-induced mitophagy in liver and FoxO3A-induced mitophagy in atrophying muscle.	Deleted, silenced or mis-localized in breast, prostate, colon, pancreatic, liver, glioma and other cancers. BNip3 loss accelerates progression to metastasis in mouse models of breast cancer and in KRas-driven PDAC.	(Chourasia et al 2015);(Glick et al 2012) (Ney 2015) (Chourasia et al 2019)
NIX (BNIP3L)	Functional homolog of BNIP3; induced by hypoxia, p53; required for mitophagy during red blood cell differentiation, retinal ganglion cell differentiation and polarization of M1 type macrophages; interacts with Rheb and LC3.	NIX knockdown promoted tumor growth in a mouse mammary tumor xenograft study. Knockout of NIX limited tumorigenesis in KRas-driven PDAC.	(Esteban-Martinez et al 2017, Fei et al 2005, Ney 2015, Sandoval et al 2008, Schweers et al 2007) (Humpton et al 2019)
Lipin-1	Lipin-1 is a phosphatidic acid phosphatase that regulates triglyceride synthesis. Lipin-1 mutation causes dysfunctional mitochondria to accumulate in muscle where Lipin-1 is required for BNIP3-dependent mitophagy.	Heritable mutations found in human myopathies; mTOR target.	(Alshudukhi et al 2018)
Rheb	Small GTPase required for mTORC1 activity; interacts with LC3 and NIX to promote mitophagy induced by switch from glycolytic to oxidative metabolism.	Rheb point mutations found in genomic analyses of kidney and endometrial cancers.	(Melser et al 2013) (Li et al 2007)
cGAS-STING/Inflammasome activation related mitophagy regulators			
Caspase-1	Proto-typical caspase involved in cytokine processing, key component	Inflammasome and IL-1 signaling promotes	(Shimada et al 2012,

Downloaded from www.annualreviews.org. University of Chicago (ar-129425) IP: 205.208.121.32 On: Tue, 22 Sept 2026 21:37:54

	of the NLRP3 inflammasome; recruited to the OMM, activated by altered $\Delta\Psi_{mt}$□□ROS and oxidized mtDNA in a positive feedback loop; cleaves Parkin.	fibrosarcoma, breast and gastric cancers.	Yu et al 2014)
NLRP3	Core component of NLRP3 inflammasome activated by DAMPs and PAMPs. Binds oxidized mtDNA released by damaged mitochondria, to activate caspase-1 and promote IL-1β secretion.	NLRP3-dependent release of IL-1β from dendritic cells is required for anti-tumor immunity.	(Zhong et al 2018) (Vanpouille-Box et al 2018)
STING	Activated by cytosolic double-stranded DNA, including mtDNA; promotes autophagosome biogenesis from the ER-Golgi, stimulates TBK1 activity that is required for OPTN and other mitophagy cargo adaptors.	Detection of cytosolic mtDNA promotes anti-tumor immunity; some human cancers exhibit reduced cGAS-STING activity. STING agonists being developed for cancer therapy.	(Chen et al 2016, Gui et al 2019) (Vanpouille-Box et al 2018)
TBK1 (TANK-binding kinase-1)	Serine/threonine kinase that phosphorylates OPTN, NDP52, TAXBP1 and p62/Sqstm1 promoting their recruitment to depolarized mitochondria in a Parkin/PINK1 dependent manner. Activated by cGAS-STING pathway in response to cytosolic dsDNA.	Mutated in ALS; required for K-Ras transformation and tumor cell survival; promotes dormancy of prostate cancer cells.	(Heo et al 2015) (Harper et al 2018)
Other			
Bcl2-L13/Bcl-Rambo	Mammalian homologue of yeast Atg32; binds LC3 through conserved LIR motif; over-expression induces mitochondrial fragmentation and mitophagy in a Parkin-independent manner.	Not known.	(Murakawa et al 2015)
Cardiolipin (CL)	IMM phospholipid involved in tethering proteins to cristae, including ETC components; mitochondrial stress induces relocalization of CL to OMM; LC3 binds to CL; CL is required for mitophagy involving LC3.	Not known.	(Chu et al 2013)
DRP-1	DRP1 is a GTPase required for mitochondrial fission and to regulate mitochondrial membrane potential. DRP1 is recruited to mitochondria by FIS1 and MFF receptors that both also promote mitophagy. DRP1 dependent fission is required for Parkin-dependent mitophagy. DRP1 plays a key role on identifying regions of the mitochondrial network	DRP1 is required for RAS transformation.	(Gomes et al 2011) (Rambold et al 2011) (Cho et al 2019)

Downloaded from www.annualreviews.org. University of Chicago (ar-129425) IP: 205.208.121.32 On: Tue, 22 Sept 2026 21:37:54

	for turnover through its interaction with ZIP1.		
EndophilinB/Bif-1	Fatty acyl transferase required for mitochondrial membrane dynamics; interacts with Beclin1 via UVRAG; colocalizes at Atg9+ puncta, involved in membrane lipid trafficking around mitochondria.	Haploinsufficiency promotes Myc-driven lymphomagenesis.	(Karbowski et al 2004, Takahashi et al 2013)
FANC-C	FANC-C localizes to mitochondria, interacts with Parkin and is required for mitophagy and to prevent inflammasome activation; role in mitophagy is genetically distinct from its role in DNA repair. Dysfunctional mitochondria accumulate in FA cells. Other FA genes also implicated in mitophagy are: FANCA, FANCD1 (BRCA2), FANCD2, FANCF, FANCL, FANCS (BRCA1).	Inactivated in Fanconi Anemia; other FANC genes include BRCA2 and BRCA1 that are deleted in hereditary breast and ovarian cancer.	(Sumpter et al 2016)
FIS1	Mitochondrial receptor for DRP1 in mitochondrial fission; phosphorylated by AMPK; required for mitophagy and self-renewal of LSCs.	Required to maintain LSCs in AML	(Pei et al 2018)
FKBP8	A member of the FK506-binding protein family localized to the OMM, possesses a LIR motif and interacts directly with LC3-related protein, LC3A recruiting it to damaged mitochondria in a Parkin-independent manner.	Not known.	(Bhujabal et al 2017)
FUNDC1	OMM protein induced by hypoxia, interacts directly with LC3; LC3 interaction regulated by ULK1, SRC, CK2, PGAM5; accumulates at ER-mito contact sites through interactions with calnexin; essential for hypoxia-induced mitophagy through recruitment of both Drp1 and LC3; interacts with MARCH5 in a ROS-dependent manner to undergo MARCH5-dependent degradation.	Not known. Knockout mouse has defects in platelet maturation.	(Chen et al 2017b, Liu et al 2012, Wu et al 2014)
MFF	Like FIS1, MFF is a mitochondrial receptor for DRP1 in mitochondrial fission. Phosphorylated by AMPK and required for fission induced by AMPK. Not clear if MFF is required for mitophagy also.	Not known.	(Toyama et al 2016)

Downloaded from www.annualreviews.org. University of Chicago (ar-129425) IP: 205.208.121.32 On: Tue, 22 Sept 2026 21:37:54

Miro	Anchors kinesins to the OMM; is phosphorylated and cleaved by Parkin/PINK1 activity resulting in reduced mitochondrial motility, possibly isolating damaged mitochondria for mitophagy; differential phosphorylation of Miro can block Parkin recruitment to OMM.	Turnover disrupted in PD.	(Hsieh et al 2016, Shlevkov et al 2016)
MTX1 (Metaxin-1)	Localizes to the OMM, possesses a non-canonical LIR and interacts with LC3C at autophagosomes to promote mitophagy independent of PINK1/Parkin. May be involved in piecemeal mitophagy.	Not known.	(Le Guerroue et al 2017)
PGAM5	Serine/threonine phosphatase at the OMM, required for CCCP-induced mitophagy, activates FUNDC1 through S13 de-phosphorylation; interacts with PINK1 at OMM in response to $\Delta\Psi_{mt}$; protects PINK1 from degradation at IMM.	Pgam5 null mice develop an age-related movement disorder, reminiscent of PD.	(Chen et al 2014)
PHB2/Prohibitin2	IMM protein involved in processing OPA1 and cristae remodeling; binds LC3 following Parkin-mediated rupture of OMM, acts as an IMM mitophagy adaptor; required for Parkin-mediated mitophagy. Requires PHB1 for stability. Also has a nuclear function regulating activity of E2F, p53 and the AR.	Expression deregulated in breast, prostate and lung cancer; role varies depending on tissue type. PHB1 deletion promotes HCC in mice.	(Wei et al 2016)
PUM2	Pumilio-2 is a RNA binding protein that inhibits protein translation. PUM-2 inhibition of MFF translation inhibited mitochondrial fission and mitophagy.	Induced during ageing in muscle and other tissues.	(D'Amico et al 2019)
SMURF	Identified in a functional screen for genes required for xenophagy; essential for Parkin-induced mitophagy; recruited to mitochondria by altered $\Delta\Psi_{mt}$; its E3 Ub ligase activity is not required for mitophagy.	Not known.	(Orvedahl et al 2011)
STX17	Syntaxin-17 is a Q-SNARE protein that promotes both autophagosome biogenesis and fusion of the autophagosome with the lysosome. Both activities involve interaction with ATG14L that it recruits to ER-mitochondrial junctions. STX17		(Hamasaki et al 2013) (Arasaki et al 2018) (Sugo et al 2018, Xian et al 2019)

	recruitment to the phagophore from the Golgi is promoted by phosphorylation by TBK1. STX17 also plays a role via PGAM5 phosphatase in determining the localization of DRP1 during mitochondrial fission and also interacts with Fis1 to promote mitophagy.		(Kumar et al 2019)
TAZ/tafazzin	Phospholipid trans-acylase that remodels cardiolipin; loss of TAZ blocks mitophagy due to failure of LC3 to recognize CL; TAZ deficiency results in reduced acylation of CL and its depletion from mitochondria.	Mutated in Barth syndrome	(Hsu et al 2015)
ZIP1	ZIP1 interacts with DRP1 at the mitochondria to modulate mitochondrial membrane potential by regulating MCU dependent uptake of $Zn2+$ ions. ZIP1 promotes $\Delta\Psi_{mt}$ and thereby controls how DRP1 induces mitophagy of depolarized mitochondria.	Not known.	(Cho et al 2019)

Downloaded from www.annualreviews.org. University of Chicago (ar-129425) IP: 205.208.121.32 On: Tue, 22 Sept 2026 21:37:54

Supplemental Table 2. Technical Approaches to Measure Mitophagy.

Technical Approach	Advantages	Disadvantages	Reference.
Electron microscopy	Provides structural information about the integrity of mitochondria, in addition to identifying mitochondria inside autophagosomes.	Only semi-quantitative. Can be difficult to locate mitochondria inside autophagosomes due to small region of cell or tissue being examined at one time.	(Chen et al 2017a, Deter & De Duve 1967)
Measure mitochondrial mass, for example by qPCR for mitochondrial genomic DNA relative to nuclear genome DNA, in the presence and absence of autophagy inhibitors, such as bafilomycin A1 or chloroquine.	Ease of use.	Only provides indirect measurement of mitophagy, is also affected by rates of biogenesis, and subject to off-target effects of lysosomotropic agents.	(Murakawa et al 2015) (Chen et al 2017a)
Measure PINK1 protein levels by western blot. PINK1 accumulates when mitochondria are depolarized.	Ease of use for cultured cells and tissue extracts.	Not all types of mitophagy rely on PINK1 or its stabilization.	(Jin et al 2018) (Chen et al 2017a)
Immunofluorescent staining to examine overlap between a constitutive mitochondrial marker (eg. TOM20) and an autophagosomal or lysosomal marker (LC3 or LAMP1). Related approach uses cells from GFP-LC3 transgenic mice and overlap with Mitotracker.	Ease of use, quantification using Image J/Fiji alogrithms.	Artifacts occur due to overlap of autophagosomes forming at mitochondria. Limited application to live cells.	(Chourasia et al 2015) (Chen et al 2017a)
mt-Keima imaging – the pH-sensitive Keima fluorophore from coral targeted using the Cox-VIII MTS to the mitochondrial matrix, exploits the alkaline nature of the mitochondrial matrix in contrast to the acid pH of the lysosome to permit detection of mitophagic flux. Keima emits fluorescence at 561 nm at acid pH and at 458 nm at alkaline pH. Mitophagic flux measured by ratiometric quantification of fluorescence at 561 nm as a proportion of total fluorescence at both wavelengths.	A direct measure of mitophagy that permits measurement of mitophagy in live cells and in tissues from mt-Keima transgenic mice, thus allowing measurement of mitophagy in genetic mouse models that delete or over-express mitophagy modulators.	Separating the two wavelengths by conventional fluorescent microscopy is technically challenging.	(Sun et al 2015) (Chen et al 2017a)
MitoQC imaging – a variation on the mCherry-GFP-LC3 autophagy reporter in which tandem mCherry-GFP is targeted to mitochondria by fusion to the MTS of the OMM protein FIS1 (or other MTS). Quenching of GFP in the acidic lysosome allows the occurrence of red mCherry-positive puncta to be taken as a measure of mitophagic flux.	Ease of use with well-characterized fluorophores; transgenic mouse expressing MitoQC, allows examination of mitophagy in mouse models that test mitophagy modulation.	Not as good *in vivo* as mt-Keima due to auto-fluorescence of some tissues.	(McWilliams et al 2016) (Chen et al 2017a)

Downloaded from www.annualreviews.org. University of Chicago (ar-129425) IP: 205.208.121.32 On: Tue, 22 Sept 2026 21:37:54

References for Tables 1 and 2.

Agnihotri S, Golbourn B, Huang X, Remke M, Younger S, et al. 2016. PINK1 Is a Negative Regulator of Growth and the Warburg Effect in Glioblastoma. *Cancer research* 76: 4708-19

Alshudukhi AA, Zhu J, Huang D, Jama A, Smith JD, et al. 2018. Lipin-1 regulates Bnip3-mediated mitophagy in glycolytic muscle. *FASEB journal : official publication of the Federation of American Societies for Experimental Biology*: fj201800374

Arasaki K, Nagashima H, Kurosawa Y, Kimura H, Nishida N, et al. 2018. MAP1B-LC1 prevents autophagosome formation by linking syntaxin 17 to microtubules. *EMBO Rep* 19

Bhujabal Z, Birgisdottir AB, Sjottem E, Brenne HB, Overvatn A, et al. 2017. FKBP8 recruits LC3A to mediate Parkin-independent mitophagy. *EMBO Rep* 18: 947-61

Chen G, Han Z, Feng D, Chen Y, Chen L, et al. 2014. A regulatory signaling loop comprising the PGAM5 phosphatase and CK2 controls receptor-mediated mitophagy. *Molecular cell* 54: 362-77

Chen L, Ma K, Han J, Chen Q, Zhu Y. 2017a. Monitoring Mitophagy in Mammalian Cells. *Methods Enzymol* 588: 187-208

Chen Q, Sun L, Chen ZJ. 2016. Regulation and function of the cGAS-STING pathway of cytosolic DNA sensing. *Nature immunology* 17: 1142-9

Chen Z, Liu L, Cheng Q, Li Y, Wu H, et al. 2017b. Mitochondrial E3 ligase MARCH5 regulates FUNDC1 to fine-tune hypoxic mitophagy. *EMBO Rep* 18: 495-509

Cho HM, Ryu JR, Jo Y, Seo TW, Choi YN, et al. 2019. Drp1-Zip1 Interaction Regulates Mitochondrial Quality Surveillance System. *Molecular cell* 73: 364-76.e8

Chourasia AH, Springer MZ, Bozek G, Poole LP, Smith AG, et al. 2019. BNIP3 promotes acinar cell homeostasis and suppresses pancreatic ductal adenocarcinoma *Under review*

Chourasia AH, Tracy K, Frankenberger C, Boland ML, Sharifi MN, et al. 2015. Mitophagy defects arising from BNip3 loss promote mammary tumor progression to metastasis. *EMBO Rep* 16: 1145-63

Chu CT, Ji J, Dagda RK, Jiang JF, Tyurina YY, et al. 2013. Cardiolipin externalization to the outer mitochondrial membrane acts as an elimination signal for mitophagy in neuronal cells. *Nat Cell Biol* 15: 1197-205

D'Amico D, Mottis A, Potenza F, Sorrentino V, Li H, et al. 2019. The RNA-Binding Protein PUM2 Impairs Mitochondrial Dynamics and Mitophagy During Aging. *Molecular cell* 73: 775-87.e10

Deter RL, De Duve C. 1967. Influence of glucagon, an inducer of cellular autophagy, on some physical properties of rat liver lysosomes. *J. Cell. Biol.* 33: 437-49

Esteban-Martinez L, Sierra-Filardi E, McGreal RS, Salazar-Roa M, Marino G, et al. 2017. Programmed mitophagy is essential for the glycolytic switch during cell differentiation. *Embo j* 36: 1688-706

Fei P, Wang W, Kim SH, Wang S, Burns TF, et al. 2005. Bnip3L is induced by p53 under hypoxia, and its knockdown promotes tumor growth. *Cancer Cell* 6: 597-609

Glick D, Zhang W, Beaton M, Marsboom G, Gruber M, et al. 2012. BNip3 regulates mitochondrial function and lipid metabolism in the liver. *Mol. Cell Biol.* 32: 2570-84

Gomes LC, Di Benedetto G, Scorrano L. 2011. During autophagy mitochondria elongate, are spared from degradation and sustain cell viability. *Nat Cell Biol* 13: 589-98

Gui X, Yang H, Li T, Tan X, Shi P, et al. 2019. Autophagy induction via STING trafficking is a primordial function of the cGAS pathway. *Nature* 567: 262-66

Hamasaki M, Furuta N, Matsuda A, Nezu A, Yamamoto A, et al. 2013. Autophagosomes form at ER-mitochondria contact sites. *Nature* 495: 389-93

Harper JW, Ordureau A, Heo JM. 2018. Building and decoding ubiquitin chains for mitophagy. *Nat Rev Mol Cell Biol* 19: 93-108

Heo JM, Ordureau A, Paulo JA, Rinehart J, Harper J. 2015. The PINK1-PARKIN Mitochondrial Ubiquitylation Pathway Drives a Program of OPTN/NDP52 Recruitment and TBK1 Activation to Promote Mitophagy. *Mol. Cell* 60: 7-20

Downloaded from www.annualreviews.org. University of Chicago (ar-129425) IP: 205.208.121.32 On: Tue, 22 Sept 2026 21:37:54

Downloaded from www.annualreviews.org. University of Chicago (ar-129425) IP: 205.208.121.32 On: Tue, 22 Sept 2026 21:37:54

Hsieh CH, Shaltouki A, Gonzalez AE, Bettencourt da Cruz A, Burbulla LF, et al. 2016. Functional Impairment in Miro Degradation and Mitophagy Is a Shared Feature in Familial and Sporadic Parkinson's Disease. *Cell Stem Cell* 19: 709-24

Hsu P, Liu X, Zhang J, Wang HG, Ye JM, Shi Y. 2015. Cardiolipin remodeling by TAZ/tafazzin is selectively required for the initiation of mitophagy. *Autophagy* 11: 643-52

Humpton TJ, Alagesan B, DeNicola G, Lu D, Yordanov GN, et al. 2019. NIX-mediated mitophagy promotes pancreatic cancer. *Under review*

Jin G, Xu C, Zhang X, Long J, Rezaeian AH, et al. 2018. Atad3a suppresses Pink1-dependent mitophagy to maintain homeostasis of hematopoietic progenitor cells. *Nature immunology* 19: 29-40

Karbowski M, Jeong SY, Youle RJ. 2004. Endophilin B1 is required for the maintenance of mitochondrial morphology. *J Cell Biol* 166: 1027-39

Kenific CM, Stehbens SJ, Goldsmith J, Leidal AM, Faure N, et al. 2016. NBR1 enables autophagy-dependent focal adhesion turnover. *J Cell Biol* 212: 577-90

Kumar S, Gu Y, Abudu YP, Bruun JA, Jain A, et al. 2019. Phosphorylation of Syntaxin 17 by TBK1 Controls Autophagy Initiation. *Dev Cell* 49: 130-44.e6

Lazarou M, Sliter DA, Kane LA, Sarraf SA, Wang C, et al. 2015. The ubiquitin kinase PINK1 recruits autophagy receptors to induce mitophagy. *Nature* 524: 309-14

Le Guerroue F, Eck F, Jung J, Starzetz T, Mittelbronn M, et al. 2017. Autophagosomal Content Profiling Reveals an LC3C-Dependent Piecemeal Mitophagy Pathway. *Molecular cell* 68: 786-96.e6

Li C, Zhang Y, Cheng X, Yuan H, Zhu S, et al. 2018. PINK1 and PARK2 Suppress Pancreatic Tumorigenesis through Control of Mitochondrial Iron-Mediated Immunometabolism. *Dev Cell* 46: 441-55.e8

Li L, Shen C, Nakamura E, Ando K, Signoretti S, et al. 2013. SQSTM1 is a pathogenic target of 5q copy number gains in kidney cancer. *Cancer Cell* 24: 738-50

Li Y, Wang Y, Kim E, Beemiller P, Wang CY, et al. 2007. Bnip3 mediates the hypoxia-induced inhibition on mTOR by interacting with Rheb. *J.Biol.Chem.* 282: 35803-13

Liu L, Feng D, Chen G, Chen M, Zheng Q, et al. 2012. Mitochondrial outer-membrane protein FUNDC1 mediates hypoxia-induced mitophagy in mammalian cells. *Nat. Cell Biol.* 14: 177-85

McWilliams TG, Prescott AR, Allen GF, Tamjar J, Munson MJ, et al. 2016. mito-QC illuminates mitophagy and mitochondrial architecture in vivo. *J Cell Biol* 214: 333-45

Melser S, Chatelain EH, Lavie J, Mahfouf W, Jose C, et al. 2013. Rheb Regulates Mitophagy Induced by Mitochondrial Energetic Status. *Cell Metab.* 17: 719-30

Moscat J, Karin M, Diaz-Meco MT. 2016. p62 in Cancer: Signaling Adaptor Beyond Autophagy. *Cell* 167: 606-09

Murakawa T, Yamaguchi O, Hashimoto A, Hikoso S, Takeda T, et al. 2015. Bcl-2-like protein 13 is a mammalian Atg32 homologue that mediates mitophagy and mitochondrial fragmentation. *Nature communications* 6: 7527

Ney PA. 2015. Mitochondrial autophagy: Origins, significance, and role of BNIP3 and NIX. *Biochim.Biophy.Acta* 1853: 2775-83

Orvedahl A, Sumpter R, Jr., Xiao G, Ng A, Zou Z, et al. 2011. Image-based genome-wide siRNA screen identifies selective autophagy factors. *Nature* 480: 113-7

Pei S, Minhajuddin M, Adane B, Khan N, Stevens BM, et al. 2018. AMPK/FIS1-Mediated Mitophagy Is Required for Self-Renewal of Human AML Stem Cells. *Cell Stem Cell* 23: 86-100.e6

Rambold AS, Kostelecky B, Elia N, Lippincott-Schwartz J. 2011. Tubular network formation protects mitochondrial from autophagosomal degradation during nutrient starvation. *Proc Natl Acad Sci U S A* 108: 10190-95

Rojansky R, Cha MY, Chan DC. 2016. Elimination of paternal mitochondria in mouse embryos occurs through autophagic degradation dependent on PARKIN and MUL1. *eLife* 5

Sandoval H, Thiagarajan P, Dasgupta SK, Scumacker A, Prchal JT, et al. 2008. Essential role for Nix in autophagic maturation of red cells. *Nature* 454: 232-5

Schweers RL, Zhang J, Randall MS, Loyd MR, Li W, et al. 2007. NIX is required for programmed mitochondrial clearance during reticulocyte maturation. *Proc. Natl. Acad. Sci. U.S.A.* 104: 19500-5
Shimada K, Crother TR, Karlin J, Dagvadorj J, Chiba N, et al. 2012. Oxidized mitochondrial DNA activates the NLRP3 inflammasome during apoptosis. *Immunity* 36: 401-14
Shlevkov E, Kramer T, Schapansky J, LaVoie MJ, Schwarz TL. 2016. Miro phosphorylation sites regulate Parkin recruitment and mitochondrial motility. *Proc Natl Acad Sci U S A* 113: E6097-e106
Sugiura A, Nagashima S, Tokuyama T, Amo T, Matsuki Y, et al. 2013. MITOL regulates endoplasmic reticulum-mitochondria contacts via Mitofusin2. *Molecular cell* 51: 20-34
Sugo M, Kimura H, Arasaki K, Amemiya T, Hirota N, et al. 2018. Syntaxin 17 regulates the localization and function of PGAM5 in mitochondrial division and mitophagy. *Embo j* 37
Sumpter R, Jr., Sirasanagandla S, Fernandez AF, Wei Y, Dong X, et al. 2016. Fanconi Anemia Proteins Function in Mitophagy and Immunity. *Cell* 165: 867-81
Sun N, Yun J, Liu J, Malide D, Liu C, et al. 2015. Measuring In Vivo Mitophagy. *Molecular cell* 60: 685-96
Takahashi Y, Hori T, Cooper TK, Liao J, Desai N, et al. 2013. Bif-1 haploinsufficiency promotes chromosomal instability and accelerates Myc-driven lymphomagenesis via suppression of mitophagy. *Blood* 121: 1622-32
Toyama EQ, Herzig S, Courchet J, Lewis TL, Jr., Loson OC, et al. 2016. Metabolism. AMP-activated protein kinase mediates mitochondrial fission in response to energy stress. *Science* 351: 275-81
Vanpouille-Box C, Demaria S, Formenti SC, Galluzzi L. 2018. Cytosolic DNA Sensing in Organismal Tumor Control. *Cancer Cell* 34: 361-78
Veeriah S, Taylor BS, Meng S, Fang F, Yilmaz E, et al. 2010. Somatic mutations of the Parkinson's disease-associated gene PARK2 in glioblastoma and other human malignancies. *Nature genetics* 42: 77-82
Villa E, Proics E, Rubio-Patino C, Obba S, Zunino B, et al. 2017. Parkin-Independent Mitophagy Controls Chemotherapeutic Response in Cancer Cells. *Cell reports* 20: 2846-59
Wei Y, Chiang WC, Sumpter R, Jr., Mishra P, Levine B. 2016. Prohibitin 2 Is an Inner Mitochondrial Membrane Mitophagy Receptor. *Cell*
Wong YC, Holzbaur EL. 2014. Optineurin is an autophagy receptor for damaged mitochondria in parkin-mediated mitophagy that is disrupted by an ALS-linked mutation. *Proc Natl Acad Sci U S A* 111: E4439-48
Wu WK, Tian W, Hu Z, Chen G, Huang L, et al. 2014. ULK1 translocates to mitochondria and phosphorylates FUNDC1 to regulate mitophagy. *EMBO Rep.* 15: 566-75
Xian H, Yang Q, Xiao L, Shen HM, Liou YC. 2019. STX17 dynamically regulated by Fis1 induces mitophagy via hierarchical macroautophagic mechanism. *Nature communications* 10: 2059
Yu JJ, Nagasu H, Murakami T, Hoang H, Broderick L, et al. 2014. Inflammasome activation leads to caspase-1 dependent mitochondrial damage and block of mitophagy. *Proc Natl Acad Sci U S A* 111: 15514-19
Yun J, Puri R, Yang H, Lizzio MA, Wu C, et al. 2014. MUL1 acts in parallel to the PINK1/parkin pathway in regulating mitofusin and compensates for loss of PINK1/parkin. *eLife* 3: e01958
Zhong Z, Liang S, Sanchez-Lopez E, He F, Shalapour S, et al. 2018. New mitochondrial DNA synthesis enables NLRP3 inflammasome activation. *Nature* 560: 198-203

Downloaded from www.annualreviews.org. University of Chicago (ar-129425) IP: 205.208.121.32 On: Tue, 22 Sept 2026 21:37:54
